# Screening Mammography Diagnostic Reference Level System According to Compressed Breast Thickness: Dubai Health

**DOI:** 10.3390/jimaging10080188

**Published:** 2024-08-05

**Authors:** Entesar Z. Dalah, Maryam K. Alkaabi, Hashim M. Al-Awadhi, Nisha A. Antony

**Affiliations:** 1 HQ Diagnostic Imaging Department, Dubai Health, Dubai 2727, United Arab Emirates; 2 College of Medicine, Mohammed Bin Rashid University, Dubai Health, Dubai 2727, United Arab Emirates; 3 Medical Imaging Department, Dubai Hospital, Dubai Health, Dubai 2727, United Arab Emirates; makalkaabi@dubaihealth.ae (M.K.A.); hmal-awadhi@dubaihealth.ae (H.M.A.-A.); nishaaantony@dubaihealth.ae (N.A.A.)

**Keywords:** screening mammography, Diagnostic Reference Levels (DRLs), United Arab Emirates (UAE), Dubai Health, bilateral mammography views

## Abstract

Screening mammography is considered to be the most effective means for the early detection of breast cancer. However, epidemiological studies suggest that longitudinal exposure to screening mammography may raise breast cancer radiation-induced risk, which begs the need for optimization and internal auditing. The present work aims to establish a comprehensive well-structured Diagnostic Reference Level (DRL) system that can be confidently used to highlight healthcare centers in need of urgent action, as well as cases exceeding the dose notification level. Screening mammographies from a total of 2048 women who underwent screening mammography at seven different healthcare centers were collected and retrospectively analyzed. The typical DRL for each healthcare center was established and defined as per (A) bilateral image view (left craniocaudal (LCC), right craniocaudal (RCC), left mediolateral oblique (LMLO), and right mediolateral oblique (RMLO)) and (B) structured compressed breast thickness (CBT) criteria. Following this, the local DRL value was established per the bilateral image views for each CBT group. Screening mammography data from a total of 8877 images were used to build this comprehensive DRL system (LCC: 2163, RCC: 2206, LMLO: 2288, and RMLO: 2220). CBTs were classified into eight groups of <20 mm, 20–29 mm, 30–39 mm, 40–49 mm, 50–59 mm, 60–69 mm, 70–79 mm, 80–89 mm, and 90–110 mm. Using the Kruskal–Wallis test, significant dose differences were observed between all seven healthcare centers offering screening mammography. The local DRL values defined per bilateral image views for the CBT group 60–69 mm were (1.24 LCC, 1.23 RCC, 1.34 LMLO, and 1.32 RMLO) mGy. The local DRL defined per bilateral image view for a specific CBT highlighted at least one healthcare center in need of optimization. Such comprehensive DRL system is efficient, easy to use, and very clinically effective.

## 1. Introduction

Breast cancer is reported as one of the leading causes of female mortality worldwide [1]. It is the most common cancer among females in the United Arab Emirates (UAE) among citizens (32.16%) and non-citizens (41.41%) [2]. To date, screening mammography has been considered to be the most effective means for breast cancer early detection [3]. Nonetheless, repeated exposure to screening mammography can lead to adverse impacts known as radiation stochastic effects [1,4,5]. Further, some women are more likely to undergo an additional number of mammography image views compared to others. This includes obese women and women with dense breasts [6]. Consequently, this increases their lifetime attributable risk of encountering radiation-induced cancer. The number of views and doses per view considerably vary among women undergoing screening mammography exams, leading to a subgroup of women receiving more than the average dose [6]. Miglioretti et al. [6] concluded that breast cancer incidence and mortality from screening mammography are affected by the radiation dose variabilities associated with the additional image views needed, breast density, and obesity. As a result, managing, tracking, and optimizing radiation exposure in screening mammography are necessities that cannot be overemphasized.

Diagnostic Reference Level (DRL) values were introduced as an optimization tool to aid in diagnostic and interventional radiology. They have been supported and recognized by the International Commission on Radiological Protection (ICRP) [7], the International Atomic Energy Agency (IAEA) [8], the World Health Organization (WHO) [9], and the UAE Federal Authority for Nuclear Regulation (FANR) [10,11]. Further to the ICRP 135 report [7], mammography DRLs can be defined using one or more dose quantities, including the incident air kerma, entrance surface air kerma, and organ dose. Today, most advanced mammography units provide an estimate of the mean glandular dose (MGD) value, which can be extracted from digital imaging and communication in medicine (DICOM) as the organ dose. MGD has been widely accepted as the most appropriate measure for predicting radiation-induced cancer risk [12].

This work aims to establish a comprehensive DRL system to optimize screening mammography radiation exposure within the Dubai Health sector, where such services are provided by several healthcare centers. The typical DRL (TDRL) values were established for each healthcare center, followed by establishing the local DRL (LDRL) value representing the entire Dubai Health sector. For optimization purposes, the TDRL and LDRL values were established for each bilateral screening mammography image view. Namely, the left craniocaudal (LCC), right craniocaudal (RCC), left mediolateral oblique (LMLO), and right mediolateral oblique (RMLO). Further, the TDRL and LDRL values were defined based on a well-structured and categorized compressed breast thickness (CBT) groups.

## 2. Material and Methods

This retrospective dose registry study was approved by our institutional scientific research ethics committee. Screening mammography dose registry data were collected over a 6-month period, from 1 November 2023 to 22 April 2024. A total of 7 healthcare centers operating under the umbrella of one healthcare sector (denoted as A, B, C, D, E, F, and G) were enrolled in this study. Center A utilizes a GE Sonography Essential mammogram unit (General Electric Healthcare), centers B, C, D, E, and G utilize a Siemens Mammomat Inspiration mammogram unit (Siemens), and center F utilizes a Siemens Mammomat Revelation (Siemens).

The GE Sonography Essential mammography unit in center A comes with a GEMS amorphous silicon digital detector that has a field of view (FOV) of 24 cm × 30.7 cm and an image matrix of 2394 × 3062. The Siemens Mammomat Inspiration (centers B, C, D, E, and G) and Revelation (center F) direct conversion systems come with the same detector specifications, a 24 cm × 30 cm Amorphous selenium (aSe) solid-state detector, 85 µm pixel size, and 2816 × 3584 image matrix.

In this screening mammography dose registry survey, we collected: (a) the total screening mammography dose that represents all possible taken bilateral views, and (b) the dose for each bilateral view separately. Herein, we considered the mammography dose quantity of the organ dose measured in mGy to establish this comprehensive DRL system. The mammography organ dose quantities for all women enrolled in this study from all 7 healthcare centers were automatically retrieved using a patient-dedicated radiation dose tracking and management platform, DOSE TQM version 19.11 (Qaelum NV, Belgium) [13]. This dose platform is linked with the Dubai Health sector’s picture archiving and communication system (PACS). The organ dose values extracted from the dose platform and DICOM tag (0040,0316) reflect the same exact values reported by the mammography unit as MGD in mGy. Therefore, the organ dose quantity will be denoted MGD throughout this work. Patient age, CBT, and mammography acquisition parameters including filter/target material, half-value layer (HVL), peak kilovoltage (kVp), current (mAs), and the focal spot size for each bilateral image view (LCC, RCC, LMLO, and RMLO) will be retrieved using our patient dose platform.

Following the ICRP report 135 [7], the LDRL value is an arbitrary notional value of a DRL quantity, set at the 75th percentile (3rd quartile) of the median distributions of the TDRL quantities obtained from the patient dose registry for the healthcare centers (A to G).

For optimization and auditing purposes, we considered classifying our sample cohort into several CBT groups that encompass the entire CBT range presented for all women enrolled in this study. The TDRL and LDRL values were established for each bilateral image view (LCC, RCC, LMLO, and RMLO) for each CBT group. A minimum of 10 cases per each bilateral image view for each CBT group were considered and used to calculate the TDRL values. No TDRL value will be reported for image views/CBT groups with less than 10 cases. The obtained TDRL values, per image view/CBT group, will then be used to calculate the LDRL values per each bilateral image view/CBT group. Ideally, a minimum of two centers will be needed to establish these LDRL values. However, LDRLs will also be considered based on one center, provided that the number of images per view per CBT group is ≥20. In this situation, the median of the entire distribution of that solo center is calculated to estimate the LDRL.

## 3. Statistical Analysis

GraphPad Prism 8, V8.03, GraphPad Software, La Jolla, CA, USA, was used to generate statistical analyses. Quantitative variables are expressed as mean, standard deviation (S.D), median, minimum (Min), maximum (Max), and 25th, 75th, and 95th percentiles. LDRL values based on CBT were established for bilateral image views (LCC, RCC, LMLO, and RMLO) to highlight the centers in need of optimization. Correlations between MGD, CBT, and patient age were analyzed using the Spearman correlation coefficient test. Statistical differences between the TDRLs of bilateral image views can be calculated using the non-parametric Kruskal–Wallis test if the dataset demonstrated a normal distribution. For a non-normal-distribution dataset, the parametric one-way ANOVA test can be used. Using the one-way ANOVA test for a non-normal-distribution dataset will result in lower statistical differences. Statistical differences between LCC and RCC, as well as LMLO and RMLO, were calculated using the *t*-Test.

## 4. Results

### 4.1. Scan Acquisition Parameters

All women enrolled in this study were scanned using full-field digital mammography units that facilitated an automatic exposure control (AEC) mode. All women were scanned with a beam focal spot of 0.3 mm, despite the image view. In addition to the AEC mode, the mammography unit in center A allowed for the automatic optimization of parameters (AOP). When enabling the AOP mode, exposure parameters including the target, filter, and kVp and mAs values were automatically adjusted and set by the mammography unit. As such, the target in the mammography unit in center A can be either Rhodium or Molybdenum. Table 1 demonstrates a descriptive summary of the scan acquisition parameters acquired by each center (A, B, C, D, E, F, and G) enrolled in this study. Scan acquisition parameters are defined per each bilateral image view (LCC, RCC, LMLO, and RMLO).

### 4.2. Patient Characteristic and Screening Mammography TDRLs and LDRL

**Figure 1:**
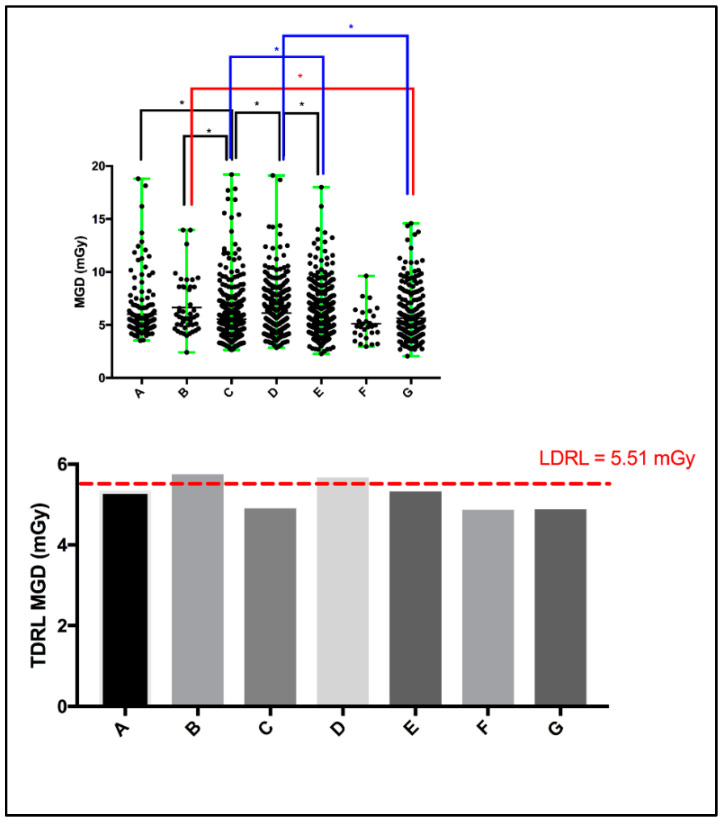
Scattered plot (top panel) demonstrating the mean and range of MGD per healthcare center, and the median MGD (i.e., TDRL) per healthcare center plotted against the calculated LDRL (bottom panel). * Significant difference, *p* < 0.05.

Screening mammography dose surveys for a total of 2048 women (including those from UAE cities in all UAE Emirates, not only Dubai, as well as non-citizens) from seven different healthcare centers (A: 183, B: 46, C: 446, D: 460, E: 644, F: 28, and G: 241) were collected and retrospectively analyzed. A scattered plot (Figure 1: top panel) shows the MGD spectrum for the entire screening mammography study per healthcare center. The MGD TDRL estimated for the entire screening mammography study is plotted against the entire screening mammography LDRL (Figure 1: bottom panel). Significant differences between healthcare centers were determined. Using the Kruskal–Wallis test, significant differences were seen between certain institutes, as denoted by * in Figure 1’s top panel. Table 2 presents a descriptive summary of the sample size (number of women enrolled), age, MGD, and CBT based on the entire screening mammography study for each healthcare center separately. The CBT ranged from 1.9 to 11.0 cm in our total cohort of 2048 patients. Accordingly, eight CBT groups were suggested to assist in dose optimization and internal auditing. The eight CBT groups were classified as: <2.0 cm, 3.0–3.9 cm, 4.0–4.9 cm, 5.0–5.9 cm, 6.0–6.9 cm, 7.0–7.9 cm, 8.0–8.9 cm, and 9.0–11.0 cm.

### 4.3. Bilateral Screening Mammography TDLRs and LDRLs

An investigation of the statistical differences between the MGD reported per each bilateral image view, LCC and RLL, and LMLO and RMLO was undertaken. Using the unpaired nonparametric *t*-test, insignificant differences were seen between LCC and RLL, as well as LMLO and RMLO.

**Figure 2:**
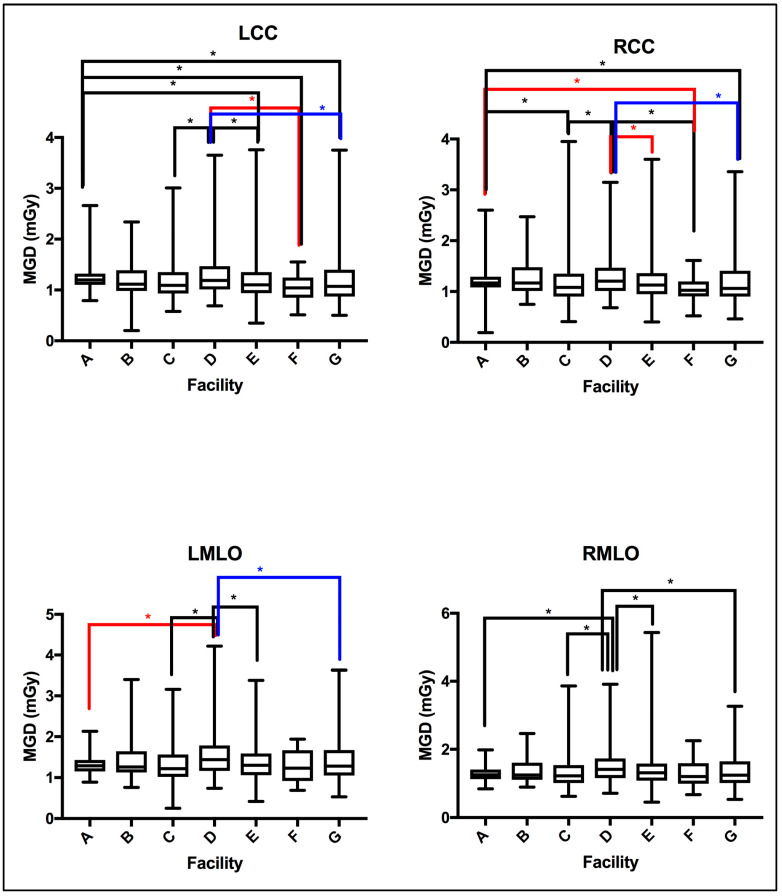
MGD TDRLs vary significantly across certain healthcare centers (facilities). Using the Kruskal–Wallis test centers showing significant difference in MGD TDRL annotated by * (*p* < 0.05).

**Figure 3:**
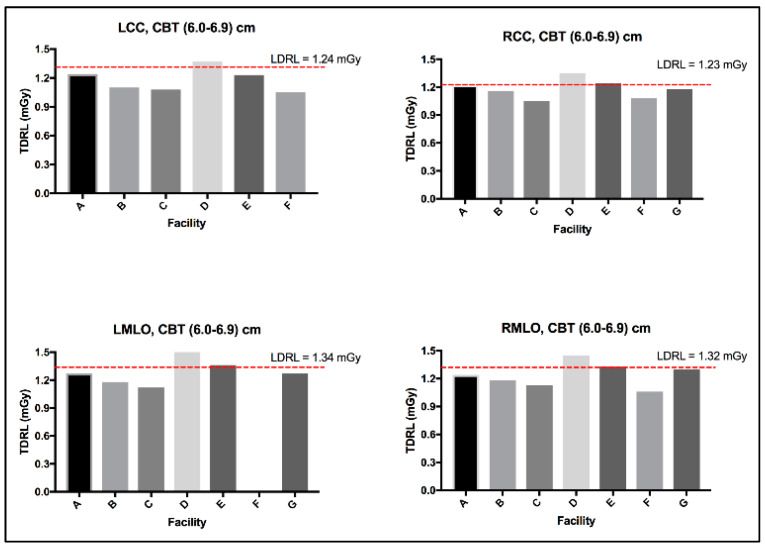
LDRL used as an aiding tool for optimization, example healthcare center D.

The MGD TDRL value for each individual center (A to G) was determined by calculating the median MGD for the entire patient cohort spectrum enrolled from each institute. Figure 2 shows the median MGD value for each institute defined as per the bilateral image views (LCC, RCC, LMLO, and RMLO). The error bars show the minimum and maximum MGD range, the line within each rectangular box represents the median MGD value (i.e., TDRL value), while the rectangular box upper and lower borders demonstrate the interquartile range (IQR: 25th to 75th percentile). An investigation of the significant differences between the MGD spectra for all enrolled centers was undertaken. Using the Kruskal–Wallis test, significant differences were seen between some centers, as annotated by *, Figure 2. The TDRL values defined for each CBT group and each bilateral image view are demonstrated in Table 3. The LDRL values per each bilateral image view (LCC, RCC, LMLO, and RMLO) for the CBTs with a minimum of 20 images were calculated as the 75th percentile of all the TDRLs, as shown in Table 4. The MGD per bilateral view for each CBT group was represented in terms of LDRL values (75th percentile of TDRL values), 25th percentile, 50th percentile, and 95th percentile. The LDRL values, as defined per the LCC, RCC, LMLO, and RMLO, were obtained for the CBT groups: 3.0–3.9 cm, 4.0–4.9 cm, 5.0–5.9 cm, 6.0–6.9 cm, 7.0–7.9 cm, and 8.0–8.9 cm. The LDRL value for CBT group 2.0–2.9 cm was only obtained for the LCC image view, while no LDRL values were obtained for CBT group 9.0–11.0 cm due to sample size limitations (less than 20 per view). The LDRL obtained per view and CBT (Table 4) will be used to highlight the healthcare centers in need of investigation, internal auditing, and optimization. One example is healthcare center D, where the TDRL of center D (all bilateral views) was shown to exceed the LDRL obtained per view for CBT group 60–69 mm, Figure 3.

### 4.4. Correlation between MGD, Age, and CBT

**Figure 4:**
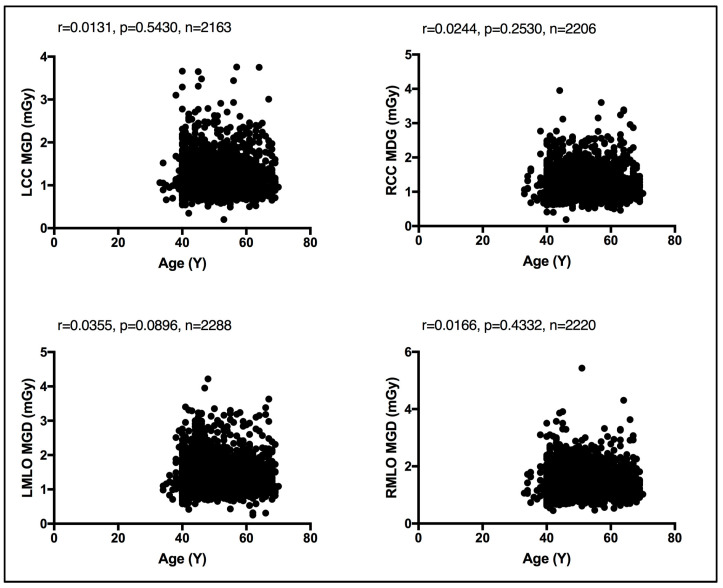
Weak, insignificant relationship between patient age and the MGD per each bilateral image view using Spearman coefficient test.

**Figure 5:**
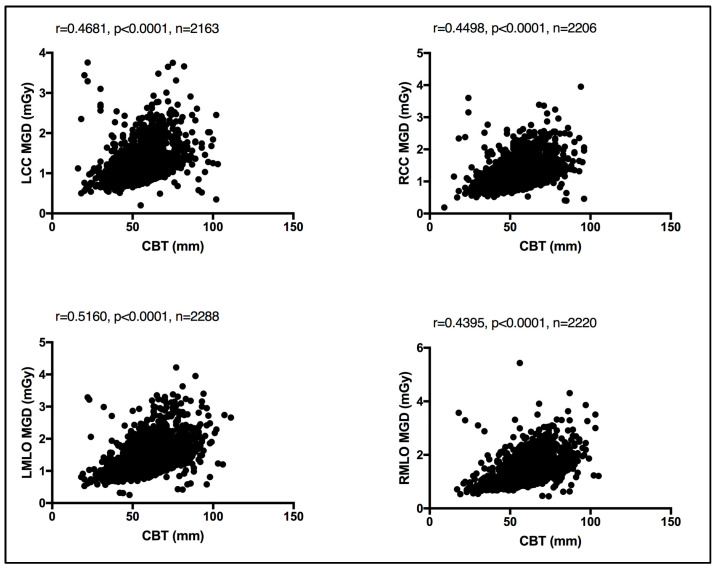
Moderate significant relationship between the CBT and MGD per each bilateral image view using Spearman coefficient test.

In the absence of breast density information, patient age was considered as a surrogate for breast density, given that breast density decreases with age [13]. An evaluation between the MGD per each bilateral image view and patient age was performed. Using the Spearman coefficient test, a weak, insignificant relationship was seen between MGD and patient age, despite the image view, as shown in Figure 4. Similarly, an evaluation of CBT was obtained for each bilateral image view. Using the Spearman coefficient test, a moderate significant relationship was seen between MGD and CBT, despite the image view, as shown in Figure 5.

## 5. Discussion

The MGD quantity is an accepted measure used to estimate radiation-induced cancer risk [5,7,14]. The present study reports the TDRL and LDRL values based on the MGD quantity.

Both breast composition (density) [15,16] and breast size [17,18] affect the amount of radiation exposure incurred from mammography. While the latter is readily available and can be automatically reported by all modern mammography units, allowing it to be exported in the form of CBT, the former, breast composition, remains a non-automated and highly subjective parameter with vast intra- and inter reader variability [19]. Herein, a weak, insignificant relationship was seen between the MGD and patient age using the Spearman coefficient test. A similar observation was reported by Dhou et al. [18] and Alahmad et al. [20]. In contrast, a significant correlation was seen between the CBT the MGD reported in this cohort of women. In line with our observation, refs. [18,20] reported the same.

The TDRL (Figure 1, bottom panel) that represents the screening mammography entire dose for each healthcare center and the LDRL value shown by the red dash-line (Figure 1 bottom panel), which represents the LDRL for screening mammography study in the Dubai healthcare sector, provide insights into the radiation exposure range across the seven healthcare centers. Both the TDRLs and LDRL demonstrated in Figure 1 and descriptively listed in Table 2 represent the total dose for screening mammography, despite the image view, number of views taken, and CBT. Clearly, center B and D show slightly higher screening mammography doses (TDRLs) compared to the remaining centers’ TDRLs. Further, center B and D’s TDRLs exceed the LDRL value established to represent screening mammography in the Dubai healthcare sector. Clinically, such insight can be effective only if the TDRL and LDRL values are defined per each bilateral image view (LCC, RCC, LMLO, and RMLO) and for several CBT groups. Screening mammography data from a total of 8877 images were used to build this comprehensive DRL system (LCC: 2163, RCC: 2206, LMLO: 2288, and RMLO: 2220). A minimum of 10 images per each bilateral image view per each CBT group were accepted to estimate the TDRL value for a particular healthcare center, as shown in Table 3. For the LDRL value, a minimum of 20 images per view per CBT group was accepted. In the scenario of a single healthcare center, the LDRL was calculated as the median. For example, the LDRL for the LCC and RCC image views with 1.44 mGy and 1.41 mGy, respectively, as demonstrated in Table 4. Studies have reported the MGD DRL based on 10 images classified by CBT cm, but not the image view [20,21]. Others have reported the MGD based on CC and MLO, averaging the bilateral LCC and RCC, as well as the LMLO and RMLO [22,23], per CBT criteria. Dhou et al. [18] and Al Naemi et al. [24] reported the MGD based on CC and MLO, with MGD being defined as per three breast density groups, with each breast density group being associated with a breast thickness range. Norsuddin et al. [23] reported the median (and range) of MGD based on bilateral image views (LCC, RCC, LMLO, and RMLO) for the entire CBT spectrum of the patients enrolled in their study, i.e., CBT was not categorized (no sub-groups), with 1.38 (0.46–5.34) mGy for RCC (CBT: 22–87 mm), 1.36 (0.72–4.53) mGy for LCC (CBT: 21–81 mm), 1.63 (0.69–6.82) mGy for RMLO (CBT: 24–99 mm), and 1.66 (0.72–6.22) mGy for RMLO (CBT: 25–99 mm).

MGD was found to significantly vary across the number of the healthcare centers within our healthcare sector, as shown in Figure 2, emphasizing the need to implement DRLs to optimize screening mammography doses. Several reasons could contribute to the MGD variation across the seven centers in Figure 2. In part, detector technology within the mammography unit may lead to slight variations in the dose [25]. The amorphous silicon flat-panel digital system used in center (A)’s mammography unit can be acquired with a lower patient radiation dose compared to the amorphous selenium flat-panel digital detector system used in centers (B to G). In addition to the type of detector used, incorporating an AOP mode (such as the one in center A) whenever available may offer a substantial dose reduction compared to just the AEC mode [26]. Further, dose variation might be affected by the CBT. Figure 3 shows a slight increase in the MGD in center D for CBT group 6.0–6.9 cm, in all image views. Improper positioning of the breast and improper breast compression may result in inconclusive mammography exams [27] on top of increasing the radiation dose [6], which often requires repeating the mammography exam, resulting in an indirect increase in patient exposure to radiation.

Existing MGDs reported at the national level, as shown in Table 5, show variations across nations’ reported MGD DRLs, implying the need for each nation to establish their own benchmark. However, comparison against existing MGD reports is challenging due to the lack of a standard MGD reporting structure. The weak, insignificant correlation between patient age and the MGD per image view demonstrated in Figure 4 suggests that age is not a dose-contributing factor. In other words, MGD does not increase or decrease with age. To the authors’ knowledge, this is the first comprehensive patient-based DRL system established for screening mammography, a system with a clear CBT structure that is defined as per bilateral image views using sufficient number of images, except for CBT group 2.0–2.9 cm. We believe that our comprehensive DRL system allows for a robust and high-confidence internal auditing process to optimize screening mammography dose. Making use of our dedicated dose monitoring and tracking platform, DOSE TQM, centers within our healthcare sector can conduct internal audits on a weekly and even daily basis. With our well-structured DRL system, the dose notification level (at the 75th percentile of the dose spectrum) can be exported to our DOSE TQM platform to flag cases exceeding a dose notification level. A limited number of patient-based studies have reported MGD DRLs using such a structured CBT that is defined per each bilateral image view. This study encountered some limitations, primarily the use of one dose quantity to establish this comprehensive DRL system. Further, the DRL system was described based on the CBT per bilateral image view only. Incorporating breast density would have added significant valuable information. MGDs attributed to diagnostic mammography views prompted as a result of abnormal screening mammographies were not included in this study. It has been suggested that the evaluation of screening mammography dose should consider the full number of image views taken in a mammography exam. Women with implants were also not included in the present study. Such women might receive double the screening mammography dose due to the need to undergo additional image views such as implant displacement views. Digital mammography implementing deep learning techniques is gaining attraction in reducing the mammography dose and lowering mammography image noise. Currently, deep learning techniques are implemented on the 3D tomosynthesis views acquired for diagnostic mammography. While our reported LDRL values were on the lower end compared to the MGD DRLs reported in the literature, as shown in Table 5, comparing the MGD LDRLs for the same CBT was a challenge.

## 6. Conclusions

DRLs were introduced as tools to aid in the optimization of radiation exposure in diagnostic and interventional radiology. However, for such tools to be clinically effective and useful, a comprehensive DRL system needs to be in place. This is particularly relevant in mammography, where the radiation dose from mammography can be, in part, affected by the image view, the number of views taken, and the CBT. The present work proposes a comprehensive DRL system that is defined using a structured CBT for screening mammography. Such a system can be used to serve in highlighting cases exceeding a dose notification level and to confidently carry out internal auditing to optimize the radiation exposure from screening mammography across healthcare sectors.

## Figures and Tables

**Table 1:** Screening mammography acquisition parameters defined per center and bilateral image view.

LCC View						
Facility	HVL	Target	Filter	CBT (cm) (Min–Max)	kVp (Min–Max)	mAs (Min–Max)
A	0.4/0.35	Rhodium/Molybdenum	Rhodium	2.5–8.9	26–31	38–131
B	0.37	Tungsten	Rhodium	3.4–9.7	27–32	17–224
C	0.37	Tungsten	Rhodium	3.0–10.2	26–33	44–276
D	0.37	Tungsten	Rhodium	1.6–8.9	26–32	45–329
E	0.37	Tungsten	Rhodium	2.0–10.3	26–32	34–322
F	0.53	Tungsten	Rhodium	4.2–7.5	28–31	42–142
G	0.37	Tungsten	Rhodium	1.8–9.3	25–31	24–496
**RCC View**						
A		Rhodium/Molybdenum	Rhodium	2.4–8.6	25–31	39–140
B	0.37	Tungsten	Rhodium	3.9–8.7	27–32	59–273
C	0.37	Tungsten	Rhodium	2.9–9.6	26–32	49–401
D	0.37	Tungsten	Rhodium	1.5–9.3	25–32	46–284
E	0.37	Tungsten	Rhodium	2.2–9.3	26–32	39–322
F	0.53	Tungsten	Rhodium	4.0–7.9	28–32	41–158
G	0.37	Tungsten	Rhodium	1.7–9.6	26–31	23–406
**LMLO View**						
A		Rhodium/Molybdenum	Rhodium	2.6–9.1	26–31	39–131
B	0.37	Tungsten	Rhodium	3.8–9.4	27–32	58–344
C	0.37	Tungsten	Rhodium	2.6–11.0	26–33	22–318
D	0.37	Tungsten	Rhodium	1.8–9.7	25–32	50–401
E	0.37	Tungsten	Rhodium	2.2–10.6	26–32	39–315
F	0.53	Tungsten	Rhodium	4.6–9.1	28–32	61–190
G	0.37	Tungsten	Rhodium	2.0–9.6	27–31	26–488
**RMLO View**						
A		Rhodium/Molybdenum	Rhodium	2.9–9.2	26–31	43–131
B	0.37	Tungsten	Rhodium	3.7–8.4	27–32	74–231
C	0.37	Tungsten	Rhodium	2.8–9.9	26–33	47–400
D	0.37	Tungsten	Rhodium	1.7–10.3	25–32	46–380
E	0.37	Tungsten	Rhodium	2.3–10.5	26–32	37–480
F	0.53	Tungsten	Rhodium	4.9–8.9	29–32	59–223
G	0.37	Tungsten	Rhodium	1.9–9.8	25–31	25–505

**Table 2:** Descriptive summary of MGDs reported per the entire mammography study for each center.

Facility	Number of Women	Age (Years)	MGD (mGy)		CBT (cm)
		Min–Max	Mean ± SD	Median (IQR)	Min–Max
A	183	33–68	6.01 ± 2.41	5.35 (4.76–5.96)	2.4–9.2
B	46	35–68	6.65 ± 2.57	5.57 (5.01–8.57)	3.4–9.7
C	446	37–70	5.55 ± 2.43	4.91 (4.05–6.16)	2.6–11.0
D	460	38–69	6.12 ± 2.10	5.67 (4.70–7.69)	1.5–10.3
E	644	34–69	5.69 ± 1.96	5.32 (4.35–6.49)	2.0–10.6
F	28	40–69	5.10 ± 1.79	4.87 (4.21–5.92)	4.0–9.1
G	241	35–69	5.63 ± 2.37	4.88 (4.04–6.43)	1.7–9.8

**Table 3:** Descriptive summary of the MGD TDRL values defined per bilateral image view and for each CBT group.

Facility	CBT (cm)	LCC View		RCC View		LMLO View		RMLO View	
		Number of Images	MGD (mGy)	Number of Images	MGD (mGy)	Number of Images	MGD (mGy)	Number of Images	MGD (mGy)
			TDRL (IQR)		TDRL (IQR)		TDRL (IQR)		TDRL (IQR)
A	4.0–4.9	23	1.00 (0.96–1.10)	23	1.06 (0.96–1.10)	8	-	8	-
	5.0–5.9	59	1.12 (1.03–1.26)	62	1.10 (1.05–1.15)	38	1.14 (1.05–1.17)	39	1.12 (1.06–1.16)
	6.0–6.9	66	1.24 (1.18–1.33)	59	1.22 (1.17–1.38)	46	1.27 (1.19–1.32)	52	1.24 (1.17–1.30)
	7.0–7.9	29	1.38 (1.31–1.44)	23	1.38 (1.28–1.46)	53	1.41 (1.32–1.49)	54	1.37 (1.29–1.45)
	8.0–8.9	2	-	5	-	19	1.56 (1.47–1.625)	16	1.54 (1.48–1.68)
B	4.0–4.9	4	-	11	1.02 (0.94–1.15)	3	-	4	-
	5.0–5.9	20	1.05 (0.95–1.24)	20	1.22 (0.96–1.36)	11	1.22 (1.00–1.74)	11	1.10 (0.98–1.15)
	6.0–6.9	21	1.10 (1.00–1.45)	15	1.16 (1.02–1.52)	11	1.18 (1.15–1.54)	17	1.18 (1.15–1.54)
	7.0–7.9	8	-	11	1.51 (1.13–1.74)	16	1.24 (1.15–1.53)	11	1.39 (1.21–1.71)
C	3.0–3.9	12	0.76 (0.67–2.04)	14	0.86 (0.75–0.99)	8	-	9	-
	4.0–4.9	42	0.96 (0.72–1.08)	45	0.87 (0.73–1.03)	40	0.88 (0.77–1.06)	34	0.91 (0.78–1.13)
	5.0–5.9	131	0.97 (0.84–1.20)	133	0.96 (0.83–1.26)	80	1.05 (0.91–1.38)	77	0.96 (0.85–1.25)
	6.0–6.9	170	1.08 (0.95–1.43)	179	1.05 (0.95–1.35)	164	1.12 (1.02–1.37)	185	1.13 (1.02–1.38)
	7.0–7.9	83	1.21 (1.09–1.39)	80	1.25 (1.13–1.54)	123	1.35 (1.19–1.71)	110	1.40 (1.23–1.84)
	8.0–8.9	23	1.44 (1.36–2.45)	21	1.41 (1.31–1.49)	50	1.49 (1.37–1.69)	58	1.50 (1.38–1.67)
	9.0–11.0	10	1.71 (1.37–2.54)	7	-	18	1.95 (1.79–2.27)	11	1.95 (1.82–2.23)
D	2.0–2.9	11	0.97 (0.81–1.09)	12	0.95 (0.83–1.19)	6	-	8	-
	3.0–3.9	55	0.89 (0.81–1.21)	54	0.91 (0.82–1.22)	32	0.95 (0.83–1.09)	27	0.92 (0.86–1.45)
	4.0–4.9	139	1.03 (0.92–1.25)	139	1.04 (0.92–1.25)	82	1.07 (0.96–1.38)	74	1.03 (0.95–1.23)
	5.0–5.9	163	1.19 (1.09–1.52)	187	1.23 (1.11–1.49)	151	1.27 (1.15–1.65)	153	1.34 (1.16–1.57)
	6.0–6.9	94	1.37 (1.26–1.63)	84	1.35 (1.26–1.55)	135	1.53 (1.35–1.88)	158	1.45 (1.30–1.83)
	7.0–7.9	15	1.43 (1.40–1.93)	19	1.62 (1.47–1.75)	69	1.68 (1.55–1.95)	76	1.69 (1.52–1.95)
	8.0–8.9	4	-	1	-	25	1.95 (1.66–2.13)	15	1.83 (1.67–1.94)
E	2.0–2.9	11	0.7 (0.63–0.96)	9	-	5	-	5	-
	3.0–3.9	39	0.80 (0.73–0.97)	35	0.80 (0.71–1.06)	25	0.83 (0.72–1.00)	25	0.88 (0.77–1.08)
	4.0–4.9	150	0.99 (0.85–1.18)	140	0.97 (0.85–1.19)	99	1.06 (0.91–1.31)	86	1.06 (0.93–1.32)
	5.0–5.9	279	1.08 (0.95–1.35)	289	1.07 (0.95–1.33)	202	1.16 (1.01–1.39)	211	1.21 (0.98–1.48)
	6.0–6.9	161	1.23 (1.10–1.51)	173	1.24 (1.10–1.52)	226	1.36 (1.19–1.66)	237	1.33 (1.17–1.62)
	7.0–7.9	38	1.36 (1.23–1.54)	48	1.32 (1.23–1.48)	114	1.50 (1.35–1.72)	114	1.43 (1.33–1.64)
	8.0–8.9	4	-	8	-	28	1.67 (1.52–1.89)	24	1.73 (1.61–1.89)
	9.0–11.0	5	-	2	-	10	1.9 (1.29–2.44)	6	-
	F	6.0–6.9	12	1.05 (0.91–1.30)	13	1.08 (0.98–1.20)	8	-	12	1.06 (0.97–1.56)
7.0–7.9		6	-	5	-	11	1.64 (1.17–1.70)	10	1.48 (1.23–1.72)
G	3.0–3.9	16	0.70 (0.62–0.84)	10	0.70 (0.64–1.00)	10	0.81 (0.69–1.29)	10	0.71 (0.68–1.00)
	4.0–4.9	38	0.78 (0.75–0.90)	39	0.83 (0.75–0.92)	21	0.95 (0.76–1.18)	18	0.94 (0.76–1.17)
	5.0–5.9	93	1.01 (0.89–1.20)	100	0.99 (0.90–1.27)	66	1.06 (0.92–1.26)	80	1.03 (0.93–1.27)
	6.0–6.9	69	1.21 (1.07–1.61)	75	1.18 (1.04–1.61)	85	1.27 (1.12–1.56)	89	1.30 (1.17–1.75)
	7.0–7.9	27	1.79 (1.39–2.13)	21	1.66 (1.41–2.45)	54	1.70 (1.45–2.17)	39	1.72 (1.48–2.26)
	8.0–8.9	4	-	6	-	12	2.22 (1.90–2.51)	12	2.11 (1.63–2.39)

CBT groups with less than 10 images in all image views were deleted. TDRLs were established for CBT groups and image views with 10 images or more.

**Table 4:** MGD LDRLs’ descriptive summary defined per bilateral image view and for each CBT group.

View	CBT (cm)	Number of Images	MGD (mGy)			
			LDRL	25th per	50th per	95th per
**LCC**	2.0–2.9	22	0.90	0.77	0.84	0.96
	3.0–3.9	122	0.82	0.75	0.78	0.88
	4.0–4.9	257	1.00	0.96	0.99	1.02
	5.0–5.9	745	1.11	1.02	1.07	1.17
	6.0–6.9	593	1.24	1.16	1.23	1.33
	7.0–7.9	192	1.43	1.36	1.38	1.72
	8.0–8.9	23	1.44 *	-	-	-
**RCC**	3.0–3.9	113	0.87	0.78	0.83	0.90
	4.0–4.9	397	1.04	0.90	1.00	1.06
	5.0–5.9	691	1.19	1.01	1.09	1.23
	6.0–6.9	498	1.23	1.12	1.18	1.32
	7.0–7.9	202	1.59	1.34	1.45	1.65
	8.9–8.9	21	1.41 *	-	-	-
**LMLO**	3.0–3.9	67	0.89	0.82	0.83	0.94
	4.0–4.9	242	1.06	0.931	1.0	1.07
	5.0–5.9	548	1.21	1.08	1.15	1.26
	6.0–6.9	667	1.34	1.20	1.27	1.49
	7.0–7.9	440	1.66	1.38	1.50	1.69
	8.0–8.9	28	1.94	1.91	1.93	1.95
**RMLO**	3.0–3.9	62	0.90	0.80	0.88	0.92
	4.0–4.9	212	1.04	0.93	0.99	1.06
	5.0–5.9	571	1.19	1.05	1.11	1.31
	6.0–6.9	750	1.32	1.16	1.24	1.41
	7.0–7.9	414	1.59	1.40	1.43	1.71
	8.0–8.9	125	1.83	1.54	1.73	2.05

* Local DRL = typical DRL value, with local DRL being calculated as the median of one healthcare facility.

**Table 5:** Present study’s MGD LDRLs in line with local and national reported DRLs.

View	CBT (cm)	Number of Images	Present Work	Literature
			MGD LDRL (mGy)	MGD DRL (mGy)
**CC**	2.0–2.9	34	0.81	
	3.0–3.9	235	0.83	1.6 (CBT: 36 mm) [28]; 3.48 (CBT: 36 mm) [29]
	4.0–4.9	654	1.02	1.10 (CBT: 4.5 ± 1.17) [30]
	5.0–5.9	1436	1.13	1.41 (CBT: 53.9 mm) [31]; 1.12 (CBT: 56.9 mm) [32]; 1.68 (CBT: 50.9 mm) [23]; 2.5 (CBT: 50–60 mm) [33]
	6.0–6.9	1091	1.23	
	7.0–7.9	394	1.49	
	8.0–8.9	44	1.43 *	
**MLO**	3.0–3.9	129	0.90	
	4.0–4.9	454	1.05	2.4 (CBT: 45 mm) [28]; 2.03 (CBT: 44 mm) [29]; 1.19 (CBT: 4.8 ± 1.22) [30]
	5.0–5.9	1119	1.18	1.48 (CBT: 53.9 mm) [31]; 1.28 (CBT: 56.9 mm) [34]; 2.25 (CBT: 58.9 mm) [23];
	6.0–6.9	1417	1.32	
	7.0–7.9	854	1.62	
	8.0–8.9	153	1.73	

* Local DRL = typical DRL value with local DRL being calculated as the median of one healthcare facility.

## Data Availability

All the data needed is presented in this paper.

## Acronyms

DRL	Diagnostic Reference Level
LCC	Left craniocaudal
RCC	Right craniocaudal
LMLO	Left mediolateral oblique
RMLO	Right mediolateral oblique
CBT	Compressed breast thickness
UAE	United Arab Emirates
ICRP	International Commission on Radiological Protection
IAEA	International Atomic Energy Agency
WHO	World Health Organization
FANR	Federal Authority for Nuclear Regulation
MGD	Mean glandular dose
DICOM	Digital imaging and communication in medicine
TDRL	Typical DRL
LDRL	Local DRL
PACS	Picture archiving and communication system
HVL	Half-value layer
kVp	Peak kilovoltage
mAs	Current
AEC	Automatic exposure control
AOP	Automatic optimization of parameters
IQR	Interquartile range
